# Psychiatric Onset of Huntington’s Disease: Clinical, Genetic and Ethical Considerations

**DOI:** 10.1192/j.eurpsy.2026.11295

**Published:** 2026-07-31

**Authors:** M. Ruiz Martínez, M. García Oñoro, M. Manjón Núñez, C. M. Moreno Delicado, K. G. Ramírez Mora, M. Sánchez Picó, C. Cuesta Moreno, C. Del Baño Hernández

**Affiliations:** 1 Psychiatry; 2 Neurology, Hospital General Universitario de Elche, Elche, Spain

## Abstract

**Introduction:**

Huntington’s disease is an autosomal dominant neurodegenerative disorder caused by the expansion of the CAG trinucleotide repeat in the *HTT* gene. It is characterised by a variable combination of motor, cognitive and psychiatric symptoms, with a mean survival of 15–20 years from diagnosis. No curative treatments are currently available. Therefore, management focuses on symptomatic care, multidisciplinary support, and genetic counselling, given its significant impact on families.

**Objectives:**

To describe the clinical course of a patient with Huntington’s disease presenting with psychiatric onset, highlighting the relevance of genetic diagnosis and counselling, as well as the role of ethics committees in clinical decision-making.

**Methods:**

We present the case of a 48-year-old man, with a suspected paternal history of Huntington’s disease, who was involuntarily admitted to the Psychiatric Inpatient Unit following a psychotic episode. During hospitalisation, clinical, psychopathological and neurological data were collected, and the patient’s competence regarding genetic testing was assessed in consultation with the Clinical Ethics Committee.

**Results:**

The patient exhibited disorganised speech, persecutory delusions, soliloquies, and behavioural disorganisation. Neurological examination revealed subtle choreic movements in the upper limbs, motor impersistence, hyperreflexia and a dysexecutive syndrome with cognitive impairment. The patient repeatedly refused genetic testing, raising an ethical dilemma related to the principles of autonomy and beneficence. The Ethics Committee concluded that he was not competent to make healthcare decisions. With family consent and judicial authorisation, genetic testing was performed, confirming one pathological allele with 42 CAG repeats and one premutated allele with 32 repeats. Treatment with risperidone achieved partial improvement of psychotic symptoms, allowing referral to the psychiatric day hospital and neurology outpatient services, with preservation of functional autonomy and participation in psychosocial rehabilitation programmes.

**Conclusions:**

This case illustrates the diagnostic and ethical complexity of Huntington’s disease when the clinical onset manifests predominantly with psychiatric symptoms. It also highlights the importance of bioethics committees in resolving clinical-ethical conflicts in clinical practice. Finally, it demonstrates the value of genetic diagnosis for clinical management, genetic counselling and planning appropriate social and healthcare resources in a progressive disease with a significant impact on autonomy and quality of life.

**Disclosure of Interest:**

M. Ruiz Martínez Employee of: I declare a direct professional relationship with the patient presented in this work, in my capacity as the treating physician. However, I have not received any funding, nor have I been subjected to any external pressure that could have influenced the preparation, interpretation, or presentation of the results., M. García Oñoro Employee of: I have not received any funding, nor have I been subjected to any external pressure that could have influenced the preparation, interpretation, or presentation of the results., M. Manjón Núñez Employee of: I have not received any funding, nor have I been subjected to any external pressure that could have influenced the preparation, interpretation, or presentation of the results., C. M. Moreno Delicado Employee of: I have not received any funding, nor have I been subjected to any external pressure that could have influenced the preparation, interpretation, or presentation of the results., K. G. Ramírez Mora Employee of: I have not received any funding, nor have I been subjected to any external pressure that could have influenced the preparation, interpretation, or presentation of the results., M. Sánchez Picó Employee of: I have not received any funding, nor have I been subjected to any external pressure that could have influenced the preparation, interpretation, or presentation of the results., C. Cuesta Moreno Employee of: I have not received any funding, nor have I been subjected to any external pressure that could have influenced the preparation, interpretation, or presentation of the results., C. Del Baño Hernández Employee of: I have not received any funding, nor have I been subjected to any external pressure that could have influenced the preparation, interpretation, or presentation of the results.

